# A homozygous *SPDYA* variant associated with meiotic arrest and failed microdissection testicular sperm extraction in non-obstructive azoospermia

**DOI:** 10.3389/fendo.2026.1916743

**Published:** 2026-09-07

**Authors:** Huimei Zheng, Yunbo Wang, Jia Wang, Weikang Chen, Jianhua Chen, Xiaohang Yang, Fan Jin, Yongmei Xi, Jingping Li

**Affiliations:** 1 Zhejiang Key Laboratory of Precision Diagnosis and Therapy for Major Gynecological Diseases, the Women's Hospital, Zhejiang University School of Medicine, Hangzhou, Zhejiang, China; 2 Center for Genetic Medicine, Zhejiang University International Institute of Medicine, Yiwu, Zhejiang, China; 3 Institute of Genetics, Zhejiang Provincial Key Laboratory of Genetic & Developmental Disorders, Zhejiang University School of Medicine, Hangzhou, Zhejiang, China; 4 Department of Reproductive Endocrinology, the Women's Hospital, Zhejiang University School of Medicine, Hangzhou, Zhejiang, China; 5 Department of Pathology, the Women's Hospital, Zhejiang University School of Medicine, Hangzhou, Zhejiang, China

**Keywords:** male infertility, meiotic arrest, micro-TESE, non-obstructive azoospermia, *SPDYA*, SUN1-SPDYA-CDK2 axis, whole-exome sequencing, XY body

## Abstract

**Background:**

Non-obstructive azoospermia (NOA) with meiotic maturation arrest is a severe form of male infertility for which genetic diagnosis may inform counseling and sperm-retrieval expectations.

**Methods:**

Whole-exome sequencing was performed in a consanguineous family with NOA, followed by *SPDYA* variant screening in 31 unrelated NOA patients and 469 men with documented reproductive outcomes. The proband underwent clinical evaluation and microdissection testicular sperm extraction (micro-TESE). Testicular histology, immunofluorescence staining, and meiotic chromosome-spread analysis were used to assess spermatogenic progression. Structural modeling, immunoblotting, co-immunoprecipitation, and exploratory bulk RNA sequencing were performed using testicular material from the proband and a pooled obstructive azoospermia control reference. The three libraries per condition were technical library replicates, not independent biological replicates.

**Results:**

A homozygous *SPDYA* missense variant (NM_182756.4:c.596G>A; p.R199H) was identified in the proband, and both parents were heterozygous carriers. No additional biallelic deleterious *SPDYA* variants were detected in the screening cohort. The patient showed NOA, reduced testicular volume, spermatocyte-stage maturation arrest, and failed micro-TESE. Cytological analyses showed absence of post-meiotic germ cells, persistent nuclear γH2AX, failed XY body formation, and no pachytene or diplotene spermatocytes. Structural modeling and co-immunoprecipitation were compatible with weakened SPDYA-CDK2 association, and immunoblotting showed lower apparent abundance of SPDYA, CDK2, and SUN1 in the patient sample. Exploratory transcriptomic profiling showed fold-change-based tissue-level trends consistent with early meiotic arrest and loss of post-meiotic germ-cell programs, rather than direct transcriptional effects of the variant.

**Conclusion:**

This single case suggests that biallelic *SPDYA* variants may be associated with poor sperm retrieval outcomes in severe meiotic arrest, but larger multicenter cohorts are strictly required before *SPDYA* can be used for clinical prediction or surgical decision-making.

## Introduction

Non-obstructive azoospermia (NOA) represents one of the most severe forms of male infertility and is characterized by impaired or absent sperm production within the testis (1). For many men with NOA, microdissection testicular sperm extraction (micro-TESE) remains the only opportunity for biological paternity; however, sperm retrieval outcomes vary substantially according to the underlying histological and genetic etiology (2, 3). Among the major histological patterns of NOA, maturation arrest (MA) is particularly relevant to genetic diagnosis because spermatogenesis is blocked at a defined developmental stage, frequently during meiotic prophase I (4). Although chromosomal abnormalities, Y-chromosome microdeletions, and monogenic defects have been implicated in NOA, a large proportion of patients with meiotic arrest remain genetically unexplained (4, 5). Recent sequencing studies have identified several meiotic genes associated with human NOA, including genes involved in homologous recombination, synapsis, and telomere-nuclear envelope attachment (5, 6), but additional disease-associated genes and variants remain to be defined.

Meiotic prophase I is a highly coordinated process during which homologous chromosomes undergo programmed DNA double-strand break formation, recombination-mediated repair, pairing, and synapsis. In male germ cells, proper organization of the X and Y chromosomes is an additional requirement. Their largely unsynapsed regions are compartmentalized into the XY body, which is marked by γH2AX and reflects appropriate meiotic sex chromosome inactivation (7, 8). These events depend in part on telomere-nuclear envelope attachment, mediated by the LINC complex (SUN1 and KASH5) together with telomere-associated factors (9–11). Disruption of this telomere-anchoring machinery impairs homologous pairing, synapsis, sex chromosome organization, and meiotic progression (9–11).

*SPDYA* (Speedy/RINGO cell cycle regulator family member A) is a meiosis-related regulator that cooperates with CDK2 during meiotic prophase (12, 13). In mice, SPDYA recruits CDK2 to telomeres and participates in the SUN1-SPDYA-CDK2 axis, which supports telomere attachment to the nuclear envelope and early meiotic progression (12–14). Loss or perturbation of components of this axis results in defective telomere dynamics, abnormal synapsis, and meiotic arrest (12, 14, 15). More recent work also suggests that SPDYA contributes to sex chromosome remodeling during pachynema (16). Despite these advances, human evidence linking *SPDYA* variants to NOA remains limited. A recently reported biallelic *SPDYA* variant supports the relevance of this gene to human meiotic arrest (16), but independent human cases and detailed clinical-pathological correlations remain scarce.

Here, we investigated *SPDYA* as a candidate gene in a consanguineous family with NOA and failed micro-TESE. We identified a homozygous *SPDYA* missense variant, p.R199H, and characterized the associated phenotype using clinical evaluation, genetic segregation analysis, testicular histology, meiotic chromosome-spread analysis, protein assays, and exploratory transcriptomic profiling. By integrating these data, we aimed to define the clinical and cytological features associated with this rare *SPDYA* variant and to evaluate its potential relevance to meiotic arrest-associated NOA.

## Materials and methods

### Study participants and ethical approval

This study was approved by the Ethics Committee of the Women’s Hospital, Zhejiang University School of Medicine (approval no. IRB-20240140-R), and written informed consent was obtained from all participants. A 27-year-old man diagnosed with NOA from a consanguineous family and his available family members were enrolled. Clinical evaluation included medical history, physical examination, semen analysis, reproductive hormone measurement, scrotal ultrasonography, karyotype analysis, and Y-chromosome AZF microdeletion screening. Semen analyses were performed according to WHO guidelines. NOA was diagnosed on the basis of azoospermia with clinical, genetic, hormonal, and testicular findings excluding obstructive causes.

Age-matched OA patients with normal spermatogenesis served as controls. The proband underwent micro-TESE. For protein and RNA assays, equivalent amounts of testicular protein or total RNA from five OA individuals were pooled to generate a common OA reference, which was not treated as five independent replicates. Detailed information on biological samples, technical replicates and quantification methods for every experiment is summarized in **Supplementary Table 1**.

### Whole-exome sequencing, variant filtering, and Sanger validation

Genomic DNA was extracted from peripheral blood. Whole-exome sequencing (WES) was performed using the Agilent SureSelect Human All Exon V6 + COSMIC kit on an Illumina HiSeq platform. Raw reads were aligned to hg19 using BWA, and variant calling was performed following GATK best-practice recommendations. Variants were annotated with ANNOVAR and prioritized based on rarity (MAF < 0.001), predicted deleteriousness, and compatibility with recessive inheritance in a consanguineous pedigree. Candidate variants were validated by Sanger sequencing in the proband and available family members. The *SPDYA* variant was described according to transcript NM_182756.4. Under the ACMG/AMP framework, the available case-level evidence is insufficient for a pathogenic or likely pathogenic classification; p.R199H is therefore conservatively regarded as a variant of uncertain significance (VUS) and a candidate disease-associated variant based on rarity and computational evidence; parental carrier status was consistent with recessive inheritance but did not provide independent cosegregation evidence. Copy-number variants (CNVs) and structural alterations were evaluated from WES BAM files using ExomeDepth (v1.1.15). The primers used for PCR amplification of *SPDYA* were as follows: forward, 5′-TTGCTTCAGTCCTATTTCCTGTT-3′; reverse, 5′-TTTTCTGTCACCCCTGCTGTA-3′.

### Population screening for *SPDYA* variants

WES data from 31 unrelated NOA patients (11 meiotic arrest, 20 with Sertoli-cell-only syndrome) were screened for SPDYA variants. Additionally, whole-genome sequencing data from 469 men with documented reproductive outcomes (natural conception, IVF, or ICSI) were analyzed as a population control dataset (National Key R&D Program of China, 2018YFC1004900). A heterozygous *SPDYA* p.Ala73Thr variant found in one Sertoli-cell-only patient was predicted to have limited functional impact by multiple *in silico* tools; it was not considered explanatory under the recessive candidate model and does not support the principal conclusion.

### Histology, immunofluorescence staining, and meiotic chromosome spreads

Testicular biopsies were fixed in Bouin’s solution, paraffin-embedded, sectioned at 5 μm, and stained with hematoxylin and eosin. For immunofluorescence, sections underwent antigen retrieval, blocking, overnight primary-antibody incubation, secondary-antibody incubation, and DAPI counterstaining. Markers included DMC1, γH2AX, CD3, CD68, PNA lectin, and TUNEL staining (**Supplementary Table 2**). Images were acquired using an Olympus FV1000 confocal microscope.

Meiotic chromosome spreads were prepared from testicular tissue and stained with antibodies against SYCP3, γH2AX, TRF1, SUN1, and CDK2 (**Supplementary Table 2**). Spermatocyte substages were classified according to SYCP3 axis morphology and γH2AX distribution. Leptotene cells were identified by short, discontinuous SYCP3-positive axial elements; zygotene cells by elongating axes with partial synapsis; pachytene cells by fully formed synaptonemal axes and stage-appropriate restriction of γH2AX to the XY body; and diplotene cells by desynapsing homologous axes. Cells designated pachytene-like (P-like) had elongated, partially synapsed SYCP3-positive axes but did not meet the full pachytene criteria, including formation of a typical γH2AX-positive XY body. The analysis was descriptive. We also clarified that all scoring was performed independently by two experienced observers. Cell and tubule counts are provided in the corresponding figure legends.

### Structural and evolutionary analysis

The potential structural effect of p.R199H on the SPDYA-CDK2 interface was evaluated using experimentally determined SPDYA-containing complex structures. The coordinates of the human SUN1-SPDYA-CDK2 ternary complex were obtained from PDB entry 7E34, and PDB entry 5UQ2 was used as an additional structural reference for the SPDYA-CDK2 interface. The experimentally determined coordinates from 7E34 were retained as the wild-type reference. The p.R199H substitution was introduced directly into the wild-type structure using the PyMOL Mutagenesis Wizard, generating a template-based *in silico* mutant structure without independent homology modeling or *de novo* structure prediction.

Candidate interface hydrogen-bond contacts were compared between the wild-type and mutant structures using an identical donor–acceptor distance cutoff of <3.5 Å. Interface contacts were inspected and visualized using PyMOL and UCSF ChimeraX. The AlphaFold-predicted monomeric SPDYA structure (AF-Q5MJ70), presented in the **Supplementary Material**, was used solely as an independent qualitative reference and was not used to construct or quantify the SPDYA-CDK2 interface. Evolutionary conservation of Arg199 across vertebrate SPDYA orthologs was assessed by multiple-sequence alignment using Clustal Omega.

### Western blotting and co-immunoprecipitation

For Western blotting, tissues were lysed in RIPA buffer (50 mM Tris-HCl pH 8.0, 150 mM NaCl, 1% IGEPAL CA-630, 0.5% sodium deoxycholate, 0.1% SDS) with protease and phosphatase inhibitors. For co-immunoprecipitation, a mild non-denaturing buffer (50 mM Tris-HCl pH 7.5, 150 mM NaCl, 1 mM EDTA, 1% Nonidet P-40) was used. Lysates were incubated on ice for 30 minutes and centrifuged at 4 °C. For co-immunoprecipitation, lysates were incubated with anti-SPDYA antibody or species-matched IgG control, and input fractions were retained. Immunoblotting was performed using antibodies against SPDYA, CDK2, SUN1, TRF1, β-tubulin, and β-actin (**Supplementary Table 2**). Band intensities were measured using ImageJ and normalized to the indicated loading controls.

### RNA sequencing, qRT-PCR, and exploratory transcriptomic analysis

Total RNA was extracted from frozen seminiferous tubules. Proband RNA and the pooled OA control RNA were each aliquoted into three equal portions, from which three sequencing libraries were prepared per sample. Libraries were sequenced on an Illumina NovaSeq 6000 platform. The RNA-seq dataset has been deposited in the Gene Expression Omnibus under accession number GSE286395. For qRT-PCR, cDNA was synthesized from available RNA samples, and amplification was performed using SYBR Green chemistry. GAPDH was used as the internal reference gene, and primer sequences are listed in **Supplementary Table 3**.

Bulk RNA-seq data were analyzed as an exploratory, fold-change-based tissue expression contrast. The three proband libraries and three pooled OA reference libraries were treated as technical RNA-seq library replicates, not independent biological replicates. Gene-level counts were normalized, and genes were ranked by log2 fold change. Functional annotation and gene set enrichment analysis were performed as hypothesis-generating analyses. Cell-type-associated signatures were assessed using marker gene sets derived from the public human testis single-cell RNA-seq dataset GSE112013. No adjusted P values or false-discovery rates were used to make inferential claims from the RNA-seq comparison.

### Statistical analysis

Histological, immunofluorescence, and meiotic chromosome-spread findings were compared descriptively between the proband and an OA control. For protein and RNA-based assays, comparisons were made between the proband and the pooled OA reference. Technical repeats, including Western blot repeats, qRT-PCR repeats, and RNA-seq library replicates, were used to assess assay reproducibility but were not treated as independent biological replicates. No inferential statistical testing was applied to single-case comparisons.

## Results

### Clinical presentation, genetic analysis, and failed micro-TESE in P01

**Figure 1:**
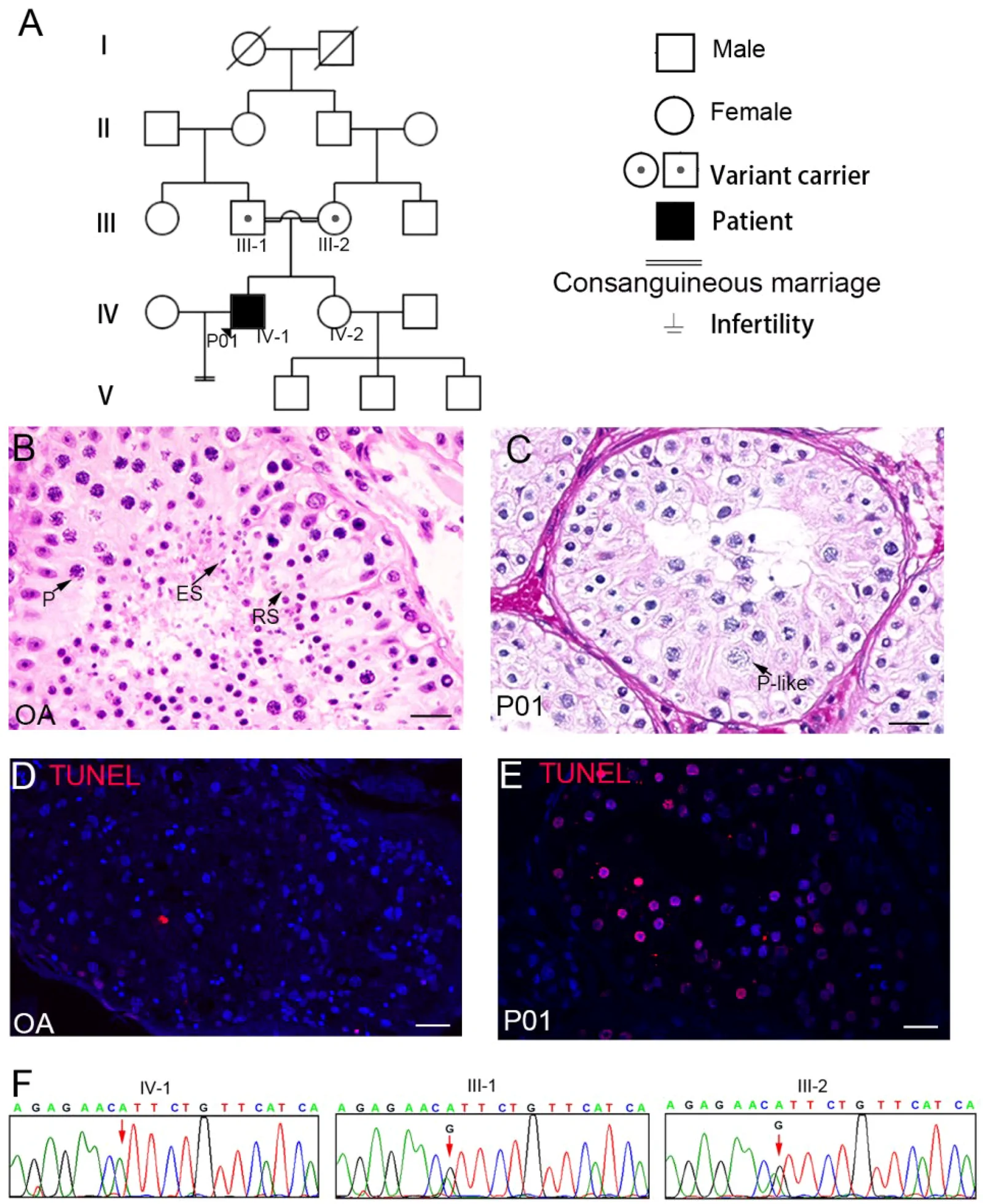
Clinical presentation, testicular histology, and genetic identification of the homozygous *SPDYA* variant in P01. **(A)** Pedigree of the consanguineous family with non-obstructive azoospermia (NOA). Double horizontal lines denote consanguinity. Squares indicate male family members and circles indicate female family members. The filled symbol indicates the affected proband. **(B, C)** Hematoxylin and eosin staining of testicular sections from an obstructive azoospermia (OA) control with normal spermatogenesis **(B)** and patient P01 **(C)**. The OA control section shows pachytene spermatocytes, round spermatids, and elongated spermatids, whereas P01 shows spermatogenic maturation arrest without morphologically identifiable post-meiotic germ cells. P, pachytene spermatocyte; RS, round spermatid; ES, elongated spermatid. Scale bar, 10 μm. **(D, E)** TUNEL staining of testicular sections from the OA control **(D)** and P01 **(E)**. TUNEL-positive cells are shown in red, and nuclei are counterstained with DAPI in blue. Scale bar, 10 μm. **(F)** Sanger sequencing validation of the *SPDYA* c.596G>A variant in available family members. The proband is homozygous for the variant, whereas both parents are heterozygous carriers.

A 27-year-old man (P01) from a consanguineous family presented with primary infertility NOA (**Figure 1A**). Repeated semen analyses showed azoospermia with normal semen volume. Reproductive hormone levels were tested twice at a 1-month interval, and their averaged levels were within the corresponding reference ranges (**Supplementary Table 4**), and genetic evaluation showed a normal 46, XY karyotype without Y-chromosome AZF microdeletions. Scrotal ultrasonography showed reduced testicular volume bilaterally (right 8.1 mL, left 7.7 mL). Micro-TESE failed to retrieve mature spermatozoa. Testicular histology showed spermatogenic maturation arrest, with seminiferous tubules containing spermatogenic cells but lacking morphologically identifiable post-meiotic germ cells or mature spermatozoa (**Figures 1B, C**). TUNEL-positive cells appeared more frequent in P01 than in OA control tissue with normal spermatogenesis (**Figures 1D, E**).

Whole-exome sequencing identified a homozygous missense variant in *SPDYA* in P01: NM_182756.4:c.596G>A, leading to p.R199H. Sanger sequencing confirmed that both parents were heterozygous carriers, consistent with segregation under an autosomal recessive model in this consanguineous family (**Figure 1F**). Systematic filtering of rare homozygous or compound heterozygous variants across the exome yielded no alternative variant showed a stronger combined fit to the phenotype and known meiotic biology (**Supplementary Table 5**). Exome-based CNV analysis revealed no structural deletions or duplications overlapping *SPDYA*. The variant was extremely rare in population databases, with an allele frequency of 0.00004456% in East Asians and 0.000004956% in gnomAD overall. Screening of 31 unrelated NOA patients and 469 men with documented reproductive outcomes did not identify additional biallelic deleterious *SPDYA* variants. Thus, the cohort did not provide independent replication. Under the ACMG/AMP framework, the available evidence does not support a pathogenic or likely pathogenic assignment; p.R199H is conservatively interpreted as a variant of uncertain significance and a candidate variant.

### *SPDYA* p.R199H is a rare homozygous candidate variant affecting a meiosis-related gene

**Figure 2:**
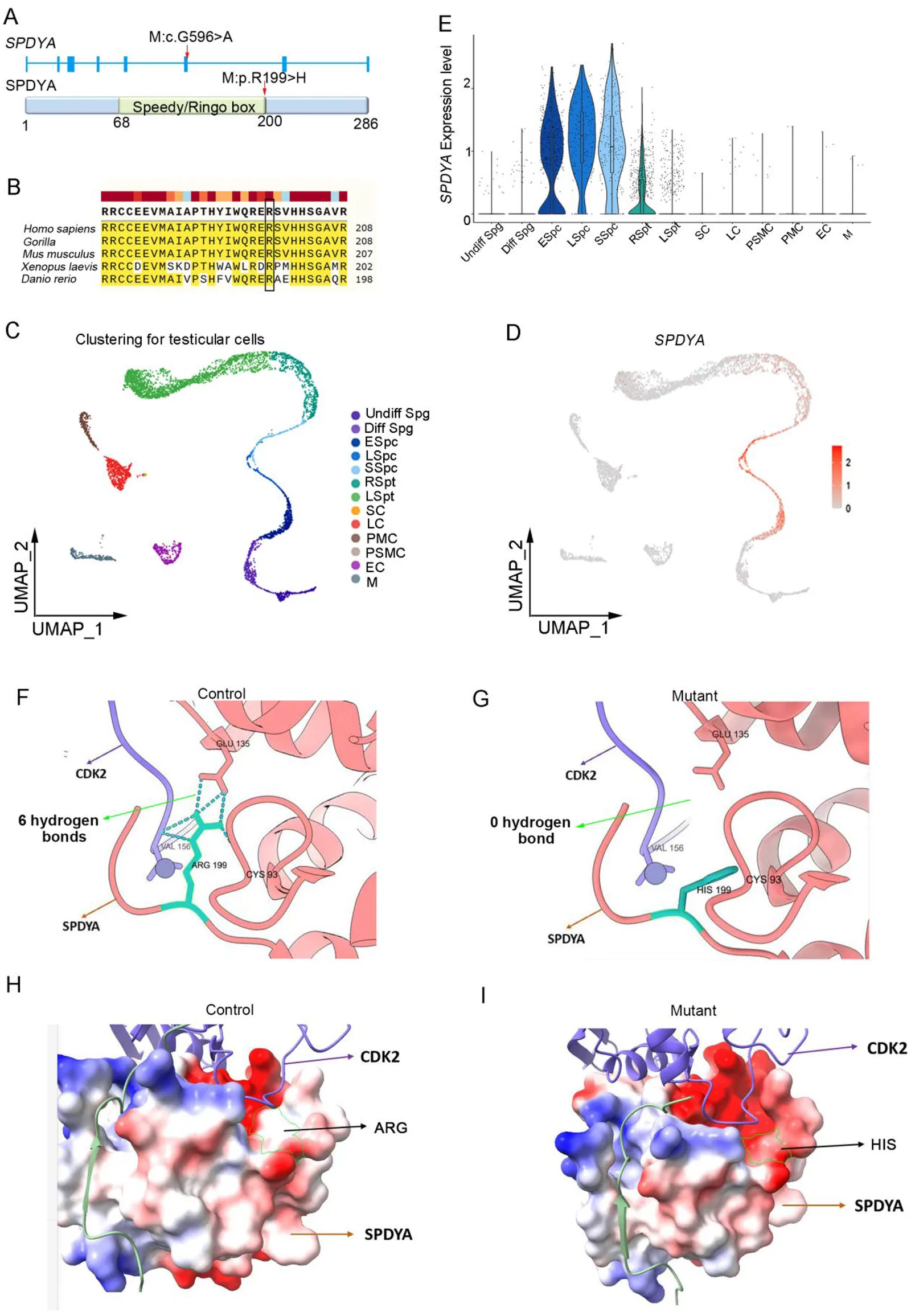
Conservation, testicular expression, and structural modeling of the *SPDYA* p.R199H variant. **(A)** Schematic representation of the *SPDYA* gene and SPDYA protein, showing the position of the identified homozygous missense variant, c.596G>A; p.R199H. **(B)** Multiple sequence alignment showing evolutionary conservation of the Arg199 residue across representative vertebrate species. The affected residue is highlighted. **(C)** UMAP projection of a public human testis single-cell RNA-seq dataset showing major germline and somatic cell populations. Undiff Spg, undifferentiated spermatogonia; Diff Spg, differentiated spermatogonia; ESpc, early spermatocytes; LSpc, late spermatocytes; SSpc, secondary spermatocytes; RSpt, round spermatids; LSpt, elongated spermatids; SC, Sertoli cells; LC, Leydig cells; PMC, peritubular myoid cells; PSMC, perivascular smooth muscle cells; EC, endothelial cells; M, macrophages. **(D)** UMAP visualization of *SPDYA* expression in the human testis single-cell RNA-seq dataset. **(E)** Violin plot showing *SPDYA* expression across testicular cell populations, with enrichment in spermatocyte populations. **(F, G)** Structural models of wild-type **(F)** and p.R199H mutant **(G)** SPDYA-CDK2 complexes. SPDYA is shown in pink and CDK2 in purple. Residue 199 is highlighted in green, showing Arg199 in the wild-type model and His199 in the mutant model. **(H, I)** Local interface views of the wild-type **(H)** and predicted mutant **(I)** SPDYA-CDK2-SUN1 complexes. CDK2 is shown in purple, SUN1 in green, and SPDYA as a surface representation. Residue 199 of SPDYA is circled. The models suggest local alteration of the SPDYA-CDK2/SUN1 interface but do not by themselves establish disrupted complex formation.

The p.R199H variant is located within the conserved Speedy/Ringo box domain (**Figure 2A**). Multiple sequence alignment showed that the affected arginine residue is highly conserved across vertebrate species (**Figure 2B**). Public human testis transcriptomic resources showed that *SPDYA* is highly enriched in the testis, and single-cell RNA-seq analysis localized *SPDYA* expression predominantly to spermatocyte populations, including early spermatocytes, late spermatocytes, and secondary spermatocytes (**Figures 2C–E**, **Supplementary Figures 1**, **2**).

Structural modeling suggested that the overall predicted structure of mutant SPDYA was broadly similar to the wild-type model, without evidence of gross structural collapse (**Supplementary Figure 3**). However, local interface analysis suggested that the wild-type Arg199 residue, together with neighboring SPDYA residues Cys93 and Glu135, contributes to hydrogen-bond interactions with CDK2 Val156 (**Figures 2F, H**). In the p.R199H model, these predicted stabilizing interactions were absent (**Figures 2G, I**). These findings support p.R199H as a rare homozygous candidate variant affecting a conserved meiosis-related protein interface.

### Cytological evidence of early meiotic arrest and failed XY body formation in P01

**Figure 3:**
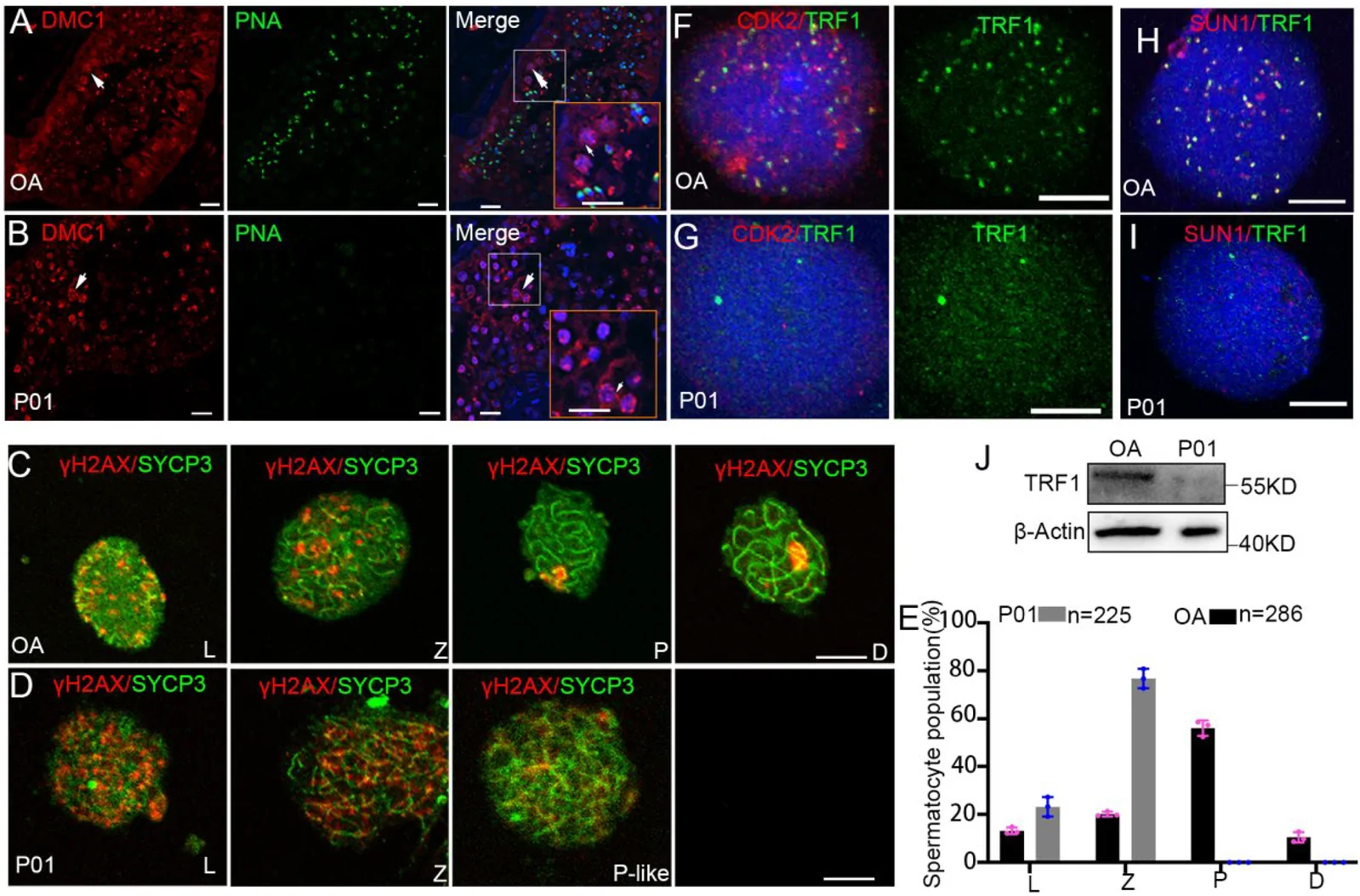
Cytological evidence of early meiotic arrest, failed XY body formation, and abnormal telomere-associated meiotic organization in P01. **(A, B)** Immunofluorescence staining of DMC1 and PNA in testicular sections from an OA control with normal spermatogenesis **(A)** and patient P01 **(B)**. DMC1, a marker associated with meiotic recombination, is shown in red. PNA, a marker of acrosome formation in spermatids and spermatozoa, is shown in green. P01 showed DMC1-positive spermatocytes but no detectable PNA-positive post-meiotic germ cells. Scale bar, 20 μm. **(C, D)** Immunofluorescence staining of meiotic chromosome spreads from the OA control **(C)** and P01 **(D)** using antibodies against SYCP3 and γH2AX. SYCP3 is shown in green and γH2AX in red. OA control spermatocytes showed stage-appropriate γH2AX localization, whereas P01 spermatocytes showed persistent nuclear-wide γH2AX staining and absence of a typical γH2AX-positive XY body. Scale bar, 10 μm. **(E)** Descriptive composition of spermatocyte substages scored from chromosome spreads of P01 and the OA control. A total of 225 spermatocytes from the single P01 and 286 spermatocytes from the single OA control were scored. L, leptotene; Z, zygotene; P-like, pachytene-like cells with elongated, partially synapsed SYCP3-positive axes that did not meet full pachytene criteria, including formation of a typical γH2AX-positive XY body; P, pachytene; D, diplotene. Percentages are shown descriptively because only one affected biological sample was available. **(F, G)** Immunofluorescence staining of spermatocyte chromosome spreads for CDK2 and TRF1 in the OA control **(F)** and P01 **(G)**. CDK2 is shown in red, TRF1 in green, and DNA in blue. Scale bar, 10 μm. **(H, I)** Immunofluorescence staining of spermatocyte chromosome spreads for SUN1 and TRF1 in the OA control **(H)** and P01 **(I)**. SUN1 is shown in red, TRF1 in green, and DNA in blue. Scale bar, 10 μm. **(J)** Immunoblot analysis of TRF1 protein abundance in testicular lysates from P01 and the OA control reference. β-actin was used as a loading control. The result is shown as descriptive protein evidence.

Having established spermatocyte-stage maturation arrest by H&E, we next performed immunofluorescence staining and meiotic chromosome-spread analysis to define the cytological features of the arrest in P01. PNA staining, which marks acrosome formation in spermatids and spermatozoa, was absent in the seminiferous tubules of P01 but readily detected in OA control tissue with normal spermatogenesis (**Figures 3A, B**).

Immunofluorescence staining for DMC1 and γH2AX further supported defective meiotic progression. DMC1-positive spermatocytes were present in P01, indicating entry into meiotic recombination-associated stages (**Figures 3A, B**). However, γH2AX showed a persistent nuclear-wide distribution in spermatocytes from P01 rather than becoming restricted to a condensed XY body, as observed in pachytene spermatocytes from OA control tissue (**Supplementary Figure 4**). This pattern suggested failure to establish the typical pachytene-stage XY body.

Chromosome-spread analysis provided more detailed cytological evidence of early meiotic arrest. Co-staining for SYCP3 and γH2AX showed that spermatocytes from P01 displayed persistent γH2AX signals across the nucleus and lacked a typical γH2AX-positive XY body (**Figures 3D, E**). In contrast, OA control spermatocytes showed γH2AX signals concentrated on XY chromatin during pachytene and diplotene stages. Descriptive scoring of 225 P01 and 286 control spermatocytes revealed no pachytene or diplotene cells in P01 (**Figure 3E**).

Because SPDYA has been implicated in the telomere-associated SUN1-SPDYA-CDK2 axis during meiosis, we also examined telomere-related markers on chromosome spreads. Co-staining of TRF1 with CDK2 or SUN1 showed abnormal telomere-associated staining patterns in P01 compared with OA control spermatocytes (**Figures 3F–I**). TRF1 signal was reduced in P01 by both chromosome-spread immunofluorescence and immunoblotting (**Figures 3F–J**). These findings indicate that the *SPDYA* p.R199H case is associated with spermatocyte-stage maturation arrest, persistent DNA-damage signaling, failed XY body formation, and abnormal telomere-associated organization.

### Exploratory protein analysis supports perturbation of the SUN1-SPDYA-CDK2 axis

**Figure 4:**
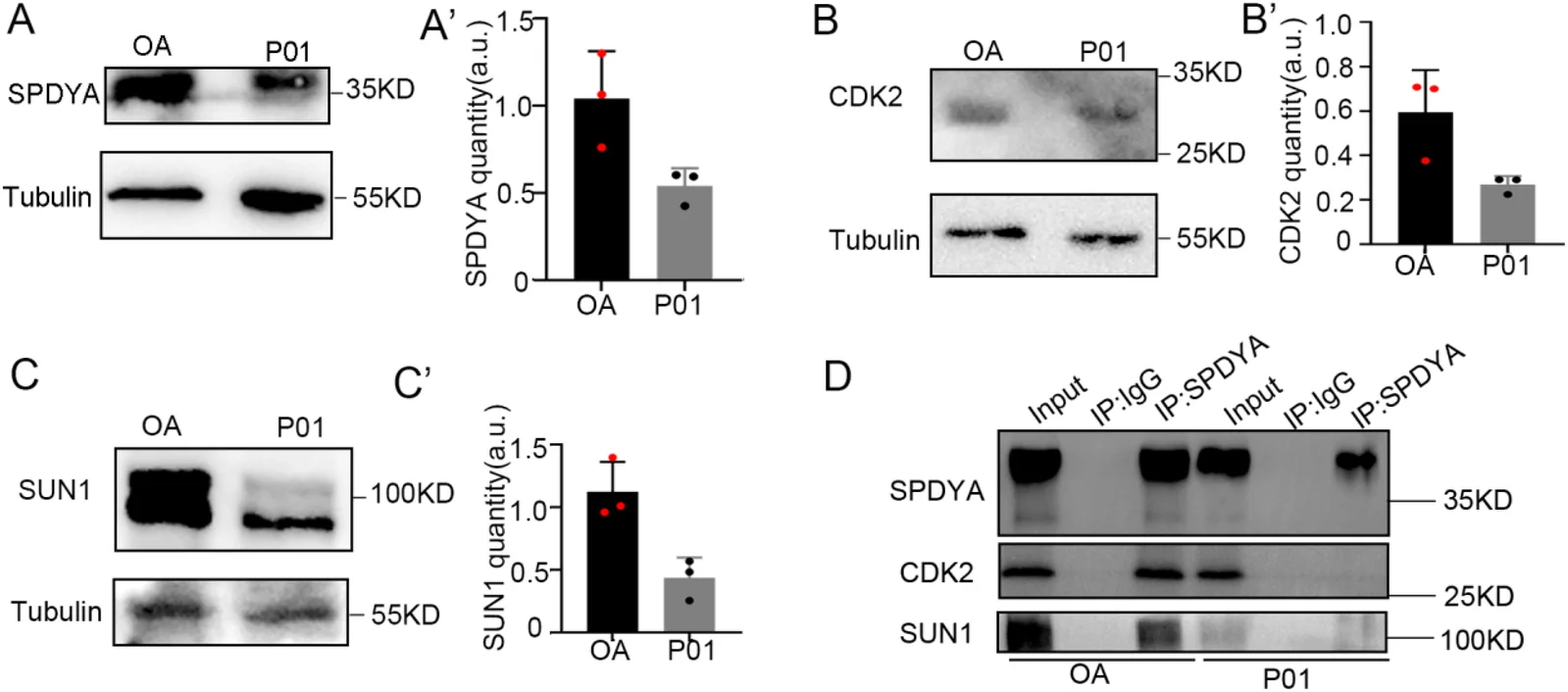
Exploratory protein analysis of the SUN1-SPDYA-CDK2 axis in P01 and one pooled OA control reference generated from five OA individuals. **(A–C)** Immunoblot analysis of SPDYA **(A)**, CDK2 **(B)**, and SUN1 **(C)** in testicular lysates from P01 and the pooled OA control reference.β-tubulin was used as the loading control, as indicated. **(A–C)** Descriptive densitometric quantification of SPDYA **(A)**, CDK2 **(B)**, and SUN1 **(C)** signals normalized to the corresponding loading control. Values were derived from technical repeats of the available biological materials and were used for descriptive quantification only; no inferential statistical testing was performed. **(D)** Co-immunoprecipitation analysis of endogenous SPDYA-associated proteins in testicular lysates from P01 and the pooled OA control reference. Immunoprecipitation was performed with anti-SPDYA antibody, followed by immunoblotting for SPDYA, CDK2, and SUN1. Input and IgG controls are shown. Reduced recovery of CDK2 and SUN1 with SPDYA in P01 is compatible with perturbation of the SUN1-SPDYA-CDK2 axis but should be interpreted as supportive biochemical evidence.

We next performed exploratory protein analyses using available testicular material from P01 and a pooled OA control reference. Immunoblotting showed lower abundance of SPDYA in P01 than in the pooled OA reference (**Figure 4A**). The apparent abundance of CDK2 and SUN1 was also lower in P01 than in the pooled OA reference (**Figures 4B, C**). Densitometric measurements were used only as descriptive quantification and were not subjected to inferential statistical testing (**Figures 4A–C**).

To examine endogenous SPDYA-associated protein recovery, co-immunoprecipitation was performed using an anti-SPDYA antibody, with input and IgG controls included. SPDYA immunoprecipitation recovered less CDK2 and SUN1 signal from the P01 testicular lysate than from the pooled OA reference (**Figure 4D**). The IgG control did not show comparable specific enrichment. These findings are compatible with weakened association of the SPDYA-CDK2/SUN1 axis in P01 testicular tissue. Because testicular tissue from NOA with meiotic arrest differs substantially in cellular composition from OA controls possessing full spermatogenesis, these lower signal intensities represent tissue-level composition trends and supportive biochemical observations.

Taken together with the structural modeling and cytological findings, the protein data support a working model in which the p.R199H variant may perturb the SUN1-SPDYA-CDK2 axis during meiotic prophase. However, because only one affected biological sample was available, these assays were used to support the candidate-gene interpretation rather than to establish causality.

### Exploratory transcriptomic profiling is consistent with loss of post-meiotic germ cells and testicular niche remodeling

**Figure 5:**
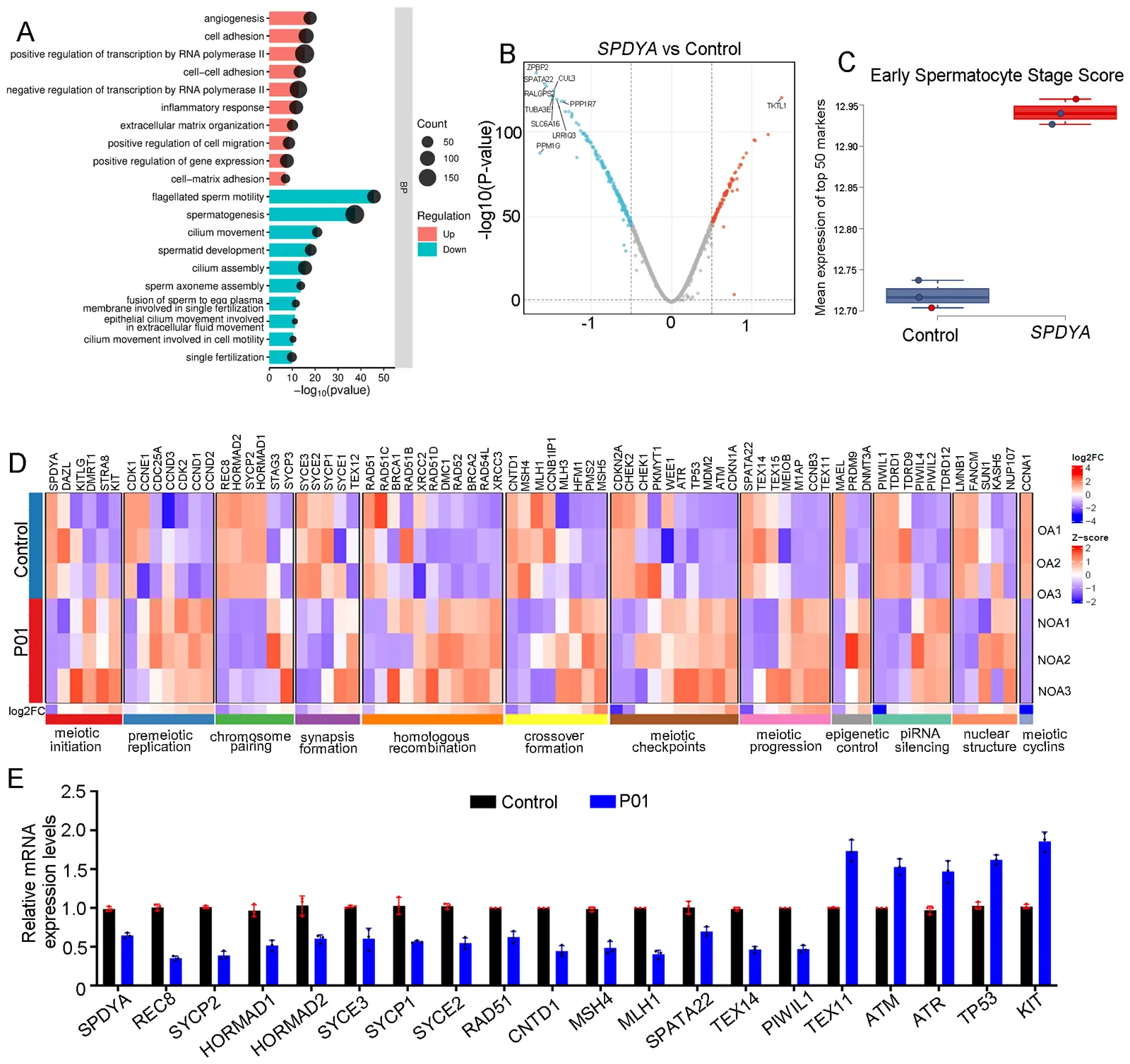
Exploratory tissue-level transcriptomic profiling in one P01 sample and one pooled OA reference. Three libraries were prepared per condition as technical library replicates; they are not independent biological replicates. **(A)** Gene Ontology annotation of genes with higher or lower measured expression in P01 versus the pooled OA reference. Genes were ranked by log_2_ fold change as a descriptive expression contrast. **(B)** Fold-change-based expression overview of 3,000 early spermatocyte-enriched genes derived from the human testis single-cell reference dataset (GSE112013). P-values are nominal values calculated from technical library replicates and are presented strictly for descriptive visualization and gene ranking. **(C)** Descriptive early spermatocyte signature score, calculated as the mean normalized expression of stage-associated markers, showing higher measured expression in P01. Individual dots denote technical library replicates. **(D)** Heatmap of selected meiosis-related genes across functional categories (cohesion, synapsis, recombination, DNA damage response, checkpoint regulation, telomere processes, and spermatogenic progression). **(E)** qRT-PCR technical measurements of selected meiotic genes; GAPDH was used as internal control. All transcriptomic analyses were exploratory and descriptive; no inferential statistical testing was applied.

The RNA-seq comparison was based on one proband sample and one pooled OA reference, with three libraries prepared from each sample as technical library replicates rather than independent biological replicates. Bulk RNA-seq showed fold-change-based trends toward lower expression of spermatogenesis-associated genes and higher expression of extracellular matrix, inflammatory, and somatic-cell-related genes in P01 (**Figure 5A**). GSEA using cell-type signatures from the Human Cell Landscape confirmed depletion of late spermatocyte and spermatid signatures and relative enrichment of spermatogonial, early spermatocyte, somatic, and immune-cell signatures in P01 (**Supplementary Figure 5**). CD68 and CD3 immunofluorescence showed greater signal in the examined P01 sections than in the OA reference sections (**Supplementary Figure 6**), without inferential statistical testing. These tissue-level patterns most likely reflect germ-cell loss and secondary tissue remodeling and should not be interpreted as a primary inflammatory mechanism.

To further examine early spermatocyte-associated changes, we analyzed a curated set of 3,000 early spermatocyte-enriched genes derived from the scRNA-seq reference. Fold-change-based visualization showed altered expression of multiple early spermatocyte transcripts in P01 (**Figure 5B**), and an early spermatocyte stage score was descriptively higher in P01 than in the pooled reference (**Figure 5C**).

Among 75 curated meiosis-related genes, 52 showed fold-change-based expression differences in P01 (**Figure 5D**). qRT-PCR technical measurements showed concordant directional trends for multiple meiotic regulators, including *SPDYA*, *REC8 *(17), *SYCP2*, *HORMAD1*, *HORMAD2 *(18), *SYCE3*, *SYCP1*, *SYCE2*, *RAD51 *(19), *CNTD1*, *MSH4*, and *MLH1 *(20), together with higher expression of *ATM*, *ATR*, and *KIT* (**Figure 5E**). All transcriptomic findings are exploratory and require validation in independent cohorts.

## Discussion

In this study, we identified a homozygous *SPDYA* missense variant, p.R199H, in a consanguineous patient with NOA, spermatocyte-stage maturation arrest, and unsuccessful micro-TESE. The variant segregated with carrier status in both parents, was extremely rare in population databases, and was not detected as a biallelic deleterious variant in additional NOA patients or controls. Together with the testis-enriched and spermatocyte-associated expression pattern of *SPDYA*, these findings support *SPDYA* p.R199H as a plausible candidate variant associated with meiotic arrest. Given the single-family design, however, this variant should be interpreted as candidate disease-associated evidence rather than definitive proof of monogenic causality.

The strongest evidence comes from the clinical and cytological phenotype. P01 showed reduced testicular volume, complete azoospermia, failed sperm retrieval by micro-TESE, and histological maturation arrest before the appearance of post-meiotic germ cells. Chromosome-spread analysis refined this defect to early meiotic prophase: P01 spermatocytes lacked typical pachytene and diplotene cells, showed persistent nuclear-wide γH2AX staining, and failed to form a condensed γH2AX-positive XY body. These findings provide a cellular explanation for the absence of retrievable mature spermatozoa in this patient.

SPDYA has been implicated in meiotic telomere biology through its cooperation with CDK2 and SUN1 (12, 13). In mice, the SUN1-SPDYA-CDK2 axis contributes to telomere attachment, homologous pairing, synapsis, and meiotic progression (14–16, 21). The p.R199H substitution lies in the conserved Speedy/Ringo box domain, and structural modeling suggested that this residue may contribute to the SPDYA-CDK2 interface. Consistently, exploratory protein analysis showed lower abundance of SPDYA, CDK2, and SUN1 in P01, and co-immunoprecipitation showed reduced recovery of CDK2 and SUN1 with SPDYA. These data are compatible with perturbation of the SUN1-SPDYA-CDK2 axis but should not be interpreted as definitive biochemical proof given the single-case design.

The cytological phenotype is also compatible with disruption of telomere-associated meiotic organization. TRF1, SUN1, and CDK2 staining on chromosome spreads suggested abnormal organization of the telomere-associated meiotic axis. Because proper telomere-nuclear envelope attachment is required for chromosome movement, homolog search, synapsis, and XY body formation (11, 22), perturbation of the SUN1-SPDYA-CDK2 axis provides a plausible working model for the early meiotic arrest observed in this case. Nevertheless, a clear limitation of this study is the lack of direct *in vitro* or *in vivo* functional assays validating the p.R199H variant. Due to the scarcity of patient testicular biopsy material, functional experiments were restricted to tissue-level protein assays. The causal sequence between p.R199H and meiotic arrest remains to be established in independent systems. Engineered cell models (*in vitro* mutant co-IP/protein stability assays in heterologous cells), animal models (gene-edited mouse models: *Spdya* R199H knock-in), or additional human cases will be required to determine whether p.R199H directly destabilizes SPDYA, weakens SPDYA-CDK2 binding, secondarily alters SUN1/CDK2 abundance, or acts through multiple mechanisms.

Interpretations of our Western blot and co-IP data must explicitly account for testicular tissue heterogeneity. OA control tissue contains abundant haploid spermatids and mature spermatozoa, whereas a meiotic-arrest testis is enriched in somatic cells and early spermatocytes while lacking post-meiotic cells. Consequently, observed reductions in SPDYA, CDK2, SUN1 may be partly driven by germ-cell depletion rather than protein instability caused by the variant. The exploratory transcriptomic data were consistent with the histological and cytological findings. P01 showed fold-change-based trends toward lower spermatogenesis-associated transcripts and higher extracellular matrix, inflammatory, and somatic-related genes, most parsimoniously explained by depletion of post-meiotic germ cells and relative enrichment of non-germline signals. Cell-type deconvolution supported this interpretation, and CD68/CD3 immunofluorescence showed higher immune-cell signals in P01, which we attribute to secondary testicular remodeling after germ cell loss instead of primary inflammation activation. However, bulk RNA-seq from a meiotic-arrest testis and a normal spermatogenesis reference cannot distinguish direct transcriptional effects of *SPDYA* disruption from secondary effects caused by germ-cell composition differences. In addition, RNA-seq libraries and repeated measurements represent technical replicates rather than independent biological replicates. Therefore, the RNA-seq data should be considered a tissue-level exploratory profile rather than evidence that *SPDYA* directly regulates the identified pathways.

From a clinical perspective, this case may be relevant to genetic counseling and surgical decision-making, but such implications must be considered speculative until validated in additional patients. The patient underwent micro-TESE, but no mature spermatozoa were retrieved, consistent with the cytological absence of post-meiotic germ cells. This supports the possibility that biallelic variants affecting early meiotic-prophase genes, including *SPDYA*, may be associated with poor sperm-retrieval outcomes. However, *SPDYA* cannot yet be considered a validated predictor of micro-TESE outcome based on a single case. The variant screening cohort of 31 NOA patients is relatively small and limits our ability to estimate the population frequency of *SPDYA* variants. Larger screening cohorts are needed to define the prevalence of SPDYA-associated meiotic arrest.

In conclusion, this study provides an additional human genotype-phenotype correlation linking a rare homozygous *SPDYA* variant with NOA, early meiotic arrest, failed XY body formation, perturbation of the SUN1-SPDYA-CDK2 axis, and unsuccessful micro-TESE. The cytological phenotype is compelling, whereas the genetic, protein, structural, and transcriptomic findings remain supportive and hypothesis-generating. *SPDYA* p.R199H should therefore be considered a candidate variant of uncertain significance associated with this phenotype, not a proven monogenic cause or a validated predictor of sperm-retrieval outcome.

## Data Availability

The datasets presented in this study can be found in online repositories. The names of the repository/repositories and accession number(s) can be found in the article/**Supplementary Material**.
