# Supplementary material for: A homozygous SPDYA variant associated with meiotic arrest and failed microdissection testicular sperm extraction in non-obstructive azoospermia
Source: Front Endocrinol (Lausanne). 2026 Sep 7;17:1916743. doi: 10.3389/fendo.2026.1916743 (PMC13590391; doi:10.3389/fendo.2026.1916743)
Supplement: Supplementary file 1 [file Supplementaryfile1.docx]

# Supplemental Table S1

**Table S1.** **Summary of biological samples and technical replicate structures per assay**

| **Assays** | Patient (P01) | Control | Control type | Technical replicates | Quantification type |
| --- | --- | --- | --- | --- | --- |
| Histology/H&E | 1 | 1OA | Individual | 3 tissue sections | Descriptive |
| Immunofluorescence | 1 | 1OA | Individual | ≥5 random microscopic fields per section | Descriptive |
| Chromosome spreads | 1 (225 cells) | 1 (286 cells) | Individual | N/A | Descriptive |
| Western blot | 1 | 5OA | Pooled | 3 repeats | Descriptive |
| Co-IP | 1 | 5OA | Pooled | 3 repeats | Descriptive |
| RNA-seq | 1 | 5OA | Pooled | 3 repeats | Descriptive |
| qRT-PCR | 1 | 5OA | Pooled | 3 repeats | Descriptive |

# Supplemental Table S2

**Table S2. Primary antibodies used in this study**

| **Antibodies** | From | Dilution rate |
| --- | --- | --- |
| **Immunofluorescence staining** | | |
| rabbit anti-DMC1 | Proteintech, 13714-1-AP | 1:500 |
| mouse anti-γH2AX | Cell Signaling Technology, 80312 | 1:1,000 |
| rabbit anti-CD3 | Proteintech, 17617-1-AP | 1:100 |
| mouse anti-CD68 | Proteintech, 66231-2-Ig | 1:500 |
| PNA | Thermo Fisher Scientific, L21409 | 1:500 |
| mouse anti-TRF1 | Abcam, ab10579 | 1:500 |
| rabbit anti-SUN1 | Novus Biologicals, NBP1-87396 | 1:300 |
| rabbit anti-SYCP3 | Proteintech, 23023-1-AP | 1:500 |
| rabbit anti-CDK2 | Proteintech, 10122-1-AP | 1:500 |
| **Western blotting** | | |
| rabbit anti-SPDYA | Novus Biologicals, NB100-2521SS | 1:1,000 |
| rabbit anti-CDK2 | Proteintech, 10122-1-AP | 1:1,000 |
| rabbit anti-SUN1 | Proteintech, 24568-1-AP | 1:1,000 |
| mouse anti-TRF1 | Proteintech, 67592-1-Ig | 1:1,000 |
| mouse anti-β-tubulin | Proteintech, 66240-1-Ig | 1:1,000 |
| mouse anti-β-actin | Proteintech, 60008-1-Ig | 1:2,000 |

# Supplemental Table S3

**Table S3. Primers** **sequences for RT-PCR**

| GAPDH-F: | 5’- CTGGGCTACACTGAGCACC-3’ |
| --- | --- |
| GAPDH-R: | 5’- AAGTGGTCGTTGAGGGCAATG-3’ |
| REC8-F: | 5’-GTGCTGGTACGAGTGCAAC-3’ |
| REC8-R: | 5’-ATAGACGCGGATCACACCGA-3’ |
| SPDYA-F: | 5’- GCCCATTACTCTGAAGCGTCC-3’ |
| SPDYA-R: | 5’- TGGGGCGTTTAGATTTGTTGTTA-3’ |
| SYCP2-F | 5’- TCGTACCTCGCATTTGTTCC-3’ |
| SYCP2-R | 5’- ATTTTCCGGGCATCTTGAGG-3’ |
| HORMAD1-F | 5’- AGCCTCCCGGTTTTAAGGATG-3’ |
| HORMAD1-R | 5’- TTCGTTCTCTCTCAGTGGTCAC-3’ |
| HORMAD2-F | 5’- TGGAGAACGCCATTTGGATG-3’ |
| HORMAD2-R | 5’- TGTCAGTACTGCCATACGTAGG-3’ |
| SYCE3-F | 5’- ATGGCCTATGACATGGTGGTG-3’ |
| SYCE3-R | 5’- TTGCCAGTTCTTCTCCATCTCC-3’ |
| SYCP1-F | 5’- TTCAGAGGGATTGAGCAGAGTG-3’ |
| SYCP1-R | 5’- ATGGCTTTTCGCTGTGCTTC-3’ |
| SYCE2-F | 5’- ATGGTGAATGCTGACAGCTG-3’ |
| SYCE2-R | 5’- TTCTCAGTGTGGCCCAAATG-3’ |
| RAD51-F | 5’- ATCGCCCATGCATCAACAAC-3’ |
| RAD51-R | 5’- ATGGCGAACATAGCTTCAGC-3’ |
| CNTD1-F | 5’- ACGAGCATGCCCTTTTTGTC-3’ |
| CNTD1-R | 5’- TGCAATGCCTGATGTTCCTC-3’ |
| MSH4-F | 5’- AGATCTCATCAACCTCGCCTTC-3’ |
| MSH4-R | 5’- TCCTGGAGTCCGAAATTGTAGC-3’ |
| MLH1-F | 5’- TGGCACAGCATCAAACCAAG-3’ |
| MLH1-R | 5’- AGCATGGCAAGGTCAAAGAG-3’ |
| SPATA22-F | 5’- AAGTTGTGTGTCGTGTTGGC-3’ |
| SPATA22-R | 5’- AGGCTTCGCTTCATTTCCTTC-3’ |
| TEX14-F | 5’- TTTGTTGCGGCGTTATTGGG-3’ |
| TEX14-R | 5’- ATTGCCCGAAAATGCTGCTG-3’ |
| PIWIL1-F | 5’- AGCAAAAGGTCACAGCAGAC-3’ |
| PIWIL1-R | 5’- TGCTGTTTGCCTAAGGTTCG-3’ |
| TEX11-F | 5’- AGCCTTTTGGTGAAGAAGCC-3’ |
| TEX11-R | 5’- TGTCACATTGCACAGCCAAG-3’ |
| ATM-F | 5’- TCAGCGAAGTGGTGTTCTTG-3’ |
| ATM-R | 5’- TTTGGCACTGAAAGGCACTG-3’ |
| TP53-F | 5’- TTGCAATAGGTGTGCGTCAG-3’ |
| TP53-R | 5’- TCCCCACAACAAAACACCAG-3’ |
| KIT-F | 5’- TCTGCTTCTGTACTGCCAGTG-3’ |
| KIT-R | 5’- ATTCAACCGTGCCATTGTGC-3’ |

# Supplemental Table S4

**Table S4. Clinical and endocrine evaluation of proband P01**

|  |  | Reference values |
| --- | --- | --- |
| **Hormone analysis^a^** | | |
| TSTO(nmol/L) | 9.91 | 8.4-28.7 |
| FSH(mIU/mL) | 6.14 | 1.4-18.1 |
| LH(mIU/mL) | 4.51 | 1.5-9.3 |

Serum hormone levels were measured on two independent clinical visits (1 month apart) and averaged.

# Supplement Figure 1

Supplementary Figure 1. *SPDYA* exhibited high expression in the human testis. The RNA-seq tissue data generated by the Genotype-Tissue Expression (GTEx) project are reported as nTPM (normalized protein-coding transcripts per million), thus corresponding to the mean values of the different individual samples from each tissue.

# Supplement Figure 2

Supplementary Figure 2. *SPDYA* showed dominant expression in the spermatocytes of human testes. The scRNA-seq data were obtained from the Human Protein Atlas (HPA) database.

# Supplement Figure 3

Supplementary Figure 3. SPDYA wild-type (WT) (green) and mutant (Mut) (blue) protein structures were predicted using the AlphaFold2 method. Protein alignment was conducted by PyMOL software after the predicted protein structures were downloaded from the AlphaFold2 service; the sites of amino acid 199 on SPDYA were highlighted red in WT and yellow in Mut.

# Supplement Figure 4

Supplementary Figure 4. (A, B) Immunofluorescent staining of γH2AX, a marker of DSB and XY body. In the proband, the results showed a pan-nuclear signal ofγH2AX signal (B), whereas a visibly distinct staining region denoting XY body could be identified in the nucleus of the OA patient (A, white arrow). Yellow arrows indicated spermatozoa in the OA patient. Bars, 20μm.

# Supplement Figure 5

Supplementary Figure 5. (A) Enrichment plots for each of the cell type show the distribution of individual genes in each set (vertical black lines). Genes sets with positive Enrichment Score indicate that they are enriched in SPDYA mutant patient whereas negative Enrichment Score indicate that they are enriched in the control OA patient. (B) Heatmap comparison of cell type-specific enriched gene sets in Control and *SPDYA* mutant. Red indicates gene sets enriched in *SPDYA* mutant and blue indicate gene sets enriched in control.

# Supplement Figure 6

Supplementary Figure 6. Descriptive immunofluorescence images of testicular sections from an OA control and patient P01 stained for (A) CD3 (marking T cells) and (B) CD68 (marking macrophages). Nuclei are counterstained with DAPI. Scale bar: 20 µm. Panels (A′) and (B′) show descriptive image-level quantification of CD3- and CD68-positive signals, respectively. Measurements obtained from multiple microscopic fields within the same individual represent technical or image-level measurements rather than independent biological replicates. Therefore, no inferential statistical testing was performed, and the results should be interpreted descriptively.
